# Differential Effects of Antihypertensive Drug Classes on Home and Office Blood Pressure Variability

**DOI:** 10.1155/ijhy/2633062

**Published:** 2026-08-02

**Authors:** Dong-Hyuk Cho, Mi-Na Kim, Jung-Joon Cha, Se Won Oh, Jae Hyoung Park, Kyung-Hee Cho, Seung Yong Shin, Eung Ju Kim, Hyung Joon Joo

**Affiliations:** ^1^ Department of Cardiology, Korea University Anam Hospital, Seoul, South Korea, kumc.or.kr; ^2^ Department of Internal Medicine, Division of Nephrology, Korea University Anam Hospital, Seoul, South Korea, kumc.or.kr; ^3^ Department of Neurology, Korea University Anam Hospital, Seoul, South Korea, kumc.or.kr; ^4^ Department of Cardiology, Korea University Ansan Hospital, Ansan, South Korea, kumc.or.kr; ^5^ Department of Cardiology, Korea University Guro Hospital, Seoul, South Korea, kumc.or.kr

## Abstract

**Objective:**

Blood pressure (BP) variability provides prognostic information beyond mean BP. However, the class‐specific associations of antihypertensive medications with BP variability remain uncertain, and few studies have directly contrasted home and office BP variability within the same patients while accounting for the white‐coat effect.

**Methods:**

In a multicenter prospective registry, patients with hypertension and adequate measurements (≥ 10 home and ≥ 5 office readings) were analyzed. The primary outcomes were systolic and diastolic average real variability (ARV) of home and office BP. Associations of renin–angiotensin system inhibitors, beta‐blockers, dihydropyridine calcium channel blockers (DHP‐CCBs), and diuretics with BP variability were estimated using inverse probability of treatment weighting (IPTW) and doubly robust models. The white‐coat effect was further adjusted using both a continuous office‐home mean difference and a guideline‐based white‐coat definition. A true‐monotherapy subcohort sensitivity analysis and class‐by‐class interaction tests were additionally performed.

**Results:**

Among 495 participants, home ARV differed minimally by antihypertensive class; a small beta‐blocker‐associated increase in home systolic BP (SBP)‐ARV was attenuated with additional adjustment. In contrast, office BP ARV was higher with DHP‐CCBs and diuretics, although the beta‐blocker association was weakened after further adjustment. Following white‐coat effect adjustment, home BP ARV remained neutral across drug classes, whereas DHP‐CCB remained associated with higher office SBP‐ARV and diuretics with higher office diastolic BP ARV. DHP‐CCBs were also associated with a lower probability of office BP control. Home BP control rates did not differ significantly by antihypertensive class.

**Conclusions:**

In this cross‐sectional observational cohort, antihypertensive medication classes showed context‐dependent associations with BP variability, which were minimal for home BP but more pronounced for office BP, partially independent of the white‐coat effect. Given potential residual confounding by indication, these hypothesis‐generating findings warrant prospective confirmation.

## 1. Introduction

Blood pressure (BP) variability is increasingly recognized as a clinically significant feature that complements mean BP in characterizing hypertension and cardiovascular prognosis [1]. Guidelines emphasize not only standard office BP monitoring but also out‐of‐office BP monitoring for diagnosis and management and provide practical threshold for home and office BP targets [2, 3]. Previous studies demonstrated that higher visit‐to‐visit and home BP variability increases stroke and cardiovascular risks independently of mean BP, suggesting BP variability as a potential therapeutic target [4, 5].

However, there is limited evidence regarding specific strategies to minimize BP variability. Current approaches for hypertension control include preferential use of long‐acting drugs that provide smoother 24‐h coverage, single‐pill combinations to improve adherence and stabilize day‐to‐day control, and self‐monitoring to guide timely titration and reduce environment‐driven fluctuations [6–8]. Despite these insights, how antihypertensive classes related to BP variability—particularly at home vs. in office—remains underexplored. Furthermore, white‐coat effect—the office BP elevation over out‐of‐office BP—may confound the BP analysis if certain antihypertensive medication class interact with clinic stress reactivity.

Using a prospective, multicenter home BP registry, we examined three previously under‐addressed questions. First, whether associations of antihypertensive medication classes with BP variability diverge between home and office settings when evaluated within the same patients. Second, whether such class‐specific associations persist after explicit adjustment for the white‐coat effect, using both a continuous office‐versus‐home mean‐difference metric and a guideline‐based binary white‐coat definition. Third, whether the observed associations are specific to short‐interval (reading‐to‐reading) variability captured by average real variability (ARV), in contrast to the visit‐to‐visit variability emphasized by previous landmark trials.

## 2. Methods

### 2.1. Study Population

This cross‐sectional analysis was conducted within a prospective, multicenter registry of home BP monitoring at three tertiary hospitals. Eligible participants were adult individuals (≥ 19 years) with a diagnosis of hypertension, who possess a home BP monitor, and at least one year of accessible electronic medical records, with anticipated medical follow‐up for a minimum of three additional years. This study adhered to the principles of the Declaration of Helsinki and was approved by the Institutional Review Board (IRB No. 2023AN0575).

Between January 2024 and June 2025, 613 patients were enrolled. A total of 519 patients had home BP recorded on ≥ 3 different days. For this analysis, we excluded patients with fewer than 10 home BP measurements or 5 office BP measurements, yielding a final sample of 495 participants. These thresholds were selected to optimize the stability of BP variability indices—including ARV, variation independent of mean (VIM), coefficient of variation (CV), and standard deviation (SD)—while maintaining adequate sample size. The ≥ 10 home BP criterion aligns with international guidelines and studies recommending ≥ 7–14 readings for reliable home BP monitoring [2, 9]. The ≥ 5 office BP threshold reflects evidence from large‐cohort and trial analyses, indicating that ≥ 5–6 visits provide stable estimates of visit‐to‐visit variability [10, 11]. BP variability was quantified on a short‐interval (reading‐to‐reading) scale for home BP (twice‐daily readings over 7 days) and on a visit‐to‐visit scale for office BP (serial in‐clinic measurements). This distinction is important because the phenotype of variability captured at different time scales may reflect different physiological and environmental determinants, and findings at one scale do not necessarily generalize to the other.

### 2.2. Home BP Monitoring

Home BP was monitored according to the recent guidelines [2, 9]. Briefly, participants were instructed to measure their BP twice daily—once in the morning and once in the evening—for seven consecutive days prior to each clinic visit. Each measurement was taken after 5 min of rest in a seated position, with the arm supported at heart level. A minimum of two readings were taken each time, with at least 1 min between readings. The home BP monitoring data were integrated into the participant’s clinical care plans. Healthcare providers could review the recorded home BP readings during clinic visits. This integration aligns with the current guidelines, which emphasize the importance of home BP monitoring in clinical practice.

### 2.3. Office BP Monitoring

Office BP was measured at each clinic visit using a validated automated oscillometric device, after a standardized resting period of at least 5 min in the seated position. Readings were obtained in a separate, quiet area rather than inside the consulting room.

### 2.4. Primary Outcome

The primary outcomes were systolic and diastolic ARV of home BP and office BP measurements, analyzed separately for systolic blood pressure (SBP) and diastolic blood pressure (DBP). ARV was selected as the primary BP variability metric because (1) it weights consecutive changes and reflects the temporal order of measurements, features that make it less sensitive than SD to slow drifts and irregular spacing [12], (2) it shows better prognostic performance than SD in several cohorts and ambulatory/home BP studies [13], and (3) it has a clear unit (mmHg) that facilitates clinical interpretation [14].

### 2.5. Statistics

Continuous variables were summarized as mean ± SD and compared with exposure groups using Student’s *t*‐test; categorical variables were presented as frequencies (%) and compared using Fisher’s exact test. Standardized mean differences (SMDs) were calculated for all baseline variables to assess covariate balance, with an absolute SMD < 0.1 considered indicative of negligible imbalance.

The primary exposure variables were four antihypertensive medication classes: renin–angiotensin system inhibitors (RAASi), beta‐blockers, dihydropyridine calcium channel blockers (DHP‐CCBs), and diuretics. Diuretic exposure included thiazide or thiazide‐like agents, loop diuretics, and potassium‐sparing agents. The full subtype distribution is presented in Table 1 and Supporting Table S1. For each exposure, propensity scores (PS) were estimated using multivariable logistic regression including prespecified baseline covariates: age, sex, diabetes mellitus, chronic kidney disease, prior myocardial infarction, prior heart failure, prior stroke, serum creatinine, total cholesterol, LDL‐C, HDL‐C, log‐transformed triglycerides, log‐transformed high‐sensitivity C‐reactive protein, non‐DHP‐CCB co‐medication, and hospital identifier. Inverse probability of treatment weighting (IPTW) was applied using the average treatment effect (ATE) formulation. Weights were stabilized by the marginal probability of treatment assignment and truncated at the 1st and 99th percentiles to reduce the influence of extreme weights. Covariate balance after weighting was examined using weighted SMDs.

**TABLE 1:** Baseline characteristics by exposure.

	RAASi				Beta‐blocker				DHP‐CCB				Diuretic			
	Exposed (*n* = 384)	Unexposed (*n* = 111)	*p* value	SMD	Exposed (*n* = 170)	Unexposed (*n* = 325)	*p* value	SMD	Exposed (*n* = 307)	Unexposed (*n* = 188)	*p* value	SMD	Exposed (*n* = 104)	Unexposed (*n* = 391)	*p* value	SMD
Age, years	57.3 ± 13.3	54.4 ± 13.9	0.05	0.21	60.8 ± 13.3	54.5 ± 13.0	< 0.01	0.48	56.0 ± 13.2	57.7 ± 13.8	0.16	−0.13	58.8 ± 14.8	56.1 ± 13.0	0.09	0.20
Male, *n* (%)	225/384 (58.6%)	65/111 (58.6%)	1.00	0.00	99/170 (58.2%)	191/325 (58.8%)	0.92	−0.01	177/307 (57.7%)	113/188 (60.1%)	0.64	−0.05	56/104 (53.8%)	234/391 (59.8%)	0.31	−0.12
Prior MI, *n* (%)	23/384 (6.0%)	7/111 (6.3%)	0.83	−0.01	14/170 (8.2%)	16/325 (4.9%)	0.17	0.13	20/307 (6.5%)	10/188 (5.3%)	0.70	0.05	8/104 (7.7%)	22/391 (5.6%)	0.49	0.08
Prior HF, *n* (%)	34/384 (8.9%)	6/111 (5.4%)	0.32	0.13	27/170 (15.9%)	13/325 (4.0%)	< 0.01	0.41	21/307 (6.8%)	19/188 (10.1%)	0.23	−0.12	31/104 (29.8%)	9/391 (2.3%)	< 0.01	0.81
Prior stroke, *n* (%)	32/384 (8.3%)	6/111 (5.4%)	0.42	0.12	12/170 (7.1%)	26/325 (8.0%)	0.86	−0.04	23/307 (7.5%)	15/188 (8.0%)	0.86	−0.02	9/104 (8.7%)	29/391 (7.4%)	0.68	0.05
DM, *n* (%)	157/384 (40.9%)	39/111 (35.1%)	0.32	0.12	91/170 (53.5%)	105/325 (32.3%)	< 0.01	0.44	133/307 (43.3%)	63/188 (33.5%)	0.04	0.20	53/104 (51.0%)	143/391 (36.6%)	0.01	0.29
CKD, *n* (%)	35/384 (9.1%)	4/111 (3.6%)	0.07	0.23	28/170 (16.5%)	11/325 (3.4%)	< 0.01	0.45	28/307 (9.1%)	11/188 (5.9%)	0.23	0.12	19/104 (18.3%)	20/391 (5.1%)	< 0.01	0.42
Statin, *n* (%)	332/384 (86.5%)	74/111 (66.7%)	< 0.01	0.48	145/170 (85.3%)	261/325 (80.3%)	0.18	0.13	263/307 (85.7%)	143/188 (76.1%)	0.01	0.25	86/104 (82.7%)	320/391 (81.8%)	0.89	0.02
OHA, *n* (%)	85/384 (22.1%)	12/111 (10.8%)	0.01	0.31	53/170 (31.2%)	44/325 (13.5%)	< 0.01	0.43	66/307 (21.5%)	31/188 (16.5%)	0.20	0.13	32/104 (30.8%)	65/391 (16.6%)	< 0.01	0.34
Insulin, *n* (%)	8/384 (2.1%)	1/111 (0.9%)	0.69	0.10	7/170 (4.1%)	2/325 (0.6%)	0.01	0.23	9/307 (2.9%)	0/188 (0.0%)	0.02	0.25	4/104 (3.8%)	5/391 (1.3%)	0.10	0.16
Creatinine (mg/dL)	0.9 ± 0.6	0.9 ± 0.2	0.06	0.14	1.0 ± 0.8	0.8 ± 0.2	< 0.01	0.37	0.9 ± 0.6	0.9 ± 0.3	0.27	0.09	1.1 ± 1.0	0.9 ± 0.3	0.05	0.39
Total cholesterol (mg/dL)	160.6 ± 38.2	162.9 ± 41.8	0.61	−0.06	154.9 ± 38.5	164.5 ± 38.9	0.01	−0.25	161.8 ± 38.1	159.9 ± 40.6	0.61	0.05	158.6 ± 37.0	161.8 ± 39.6	0.43	−0.08
LDL‐cholesterol (mg/dL)	86.8 ± 35.4	88.9 ± 35.0	0.60	−0.06	82.4 ± 33.3	89.9 ± 36.2	0.02	−0.21	87.2 ± 35.7	87.4 ± 34.7	0.95	−0.01	87.1 ± 36.9	87.3 ± 34.9	0.96	−0.01
HDL‐cholesterol (mg/dL)	51.1 ± 13.1	51.3 ± 11.1	0.86	−0.02	47.9 ± 10.6	53.0 ± 13.4	< 0.01	−0.40	51.2 ± 12.7	51.1 ± 12.8	0.93	0.01	48.6 ± 11.4	51.9 ± 13.0	0.02	−0.25
Triglyceride (mg/dL)	138.5 ± 96.4	132.1 ± 72.9	0.47	0.07	139.1 ± 83.2	136.1 ± 96.5	0.72	0.03	137.9 ± 84.3	135.8 ± 103.8	0.82	0.02	146.7 ± 105.7	134.5 ± 87.6	0.28	0.13
hsCRP (mg/L)	1.5 ± 1.9	2.0 ± 2.3	0.08	−0.23	1.7 ± 1.9	1.6 ± 2.0	0.82	0.02	1.8 ± 2.2	1.3 ± 1.6	0.01	0.23	1.8 ± 2.3	1.6 ± 1.9	0.38	0.11
Home SBP (mmHg)	123.8 ± 10.0	122.8 ± 8.3	0.26	0.11	124.5 ± 10.5	123.2 ± 9.1	0.17	0.14	124.1 ± 9.6	122.8 ± 9.6	0.13	0.14	122.9 ± 11.6	123.8 ± 9.0	0.49	−0.09
Home DBP (mmHg)	78.1 ± 8.6	78.8 ± 8.2	0.46	−0.08	76.4 ± 9.5	79.2 ± 7.8	< 0.01	−0.33	78.5 ± 8.3	77.8 ± 8.9	0.36	0.09	77.1 ± 9.8	78.6 ± 8.1	0.15	−0.18
Home BP count	83.7 ± 127.8	86.0 ± 104.8	0.84	−0.02	90.8 ± 147.1	80.8 ± 108.3	0.44	0.08	82.0 ± 132.2	87.8 ± 106.3	0.59	−0.05	81.7 ± 92.9	84.9 ± 129.9	0.78	−0.03
Office SBP (mmHg)	136.6 ± 12.8	138.1 ± 12.7	0.31	−0.12	136.4 ± 13.5	137.2 ± 12.4	0.53	−0.06	138.6 ± 12.2	133.7 ± 13.4	< 0.01	0.39	135.4 ± 15.2	137.3 ± 12.1	0.25	−0.15
Office DBP (mmHg)	82.1 ± 10.2	84.8 ± 9.9	0.03	−0.26	80.4 ± 10.5	83.9 ± 9.8	< 0.01	−0.36	83.4 ± 9.7	81.3 ± 10.9	0.04	0.21	80.6 ± 11.4	83.2 ± 9.8	0.05	−0.26
Office BP count	16.8 ± 19.1	12.7 ± 10.4	0.01	0.23	21.0 ± 24.4	13.1 ± 11.5	< 0.01	0.46	16.8 ± 19.5	14.5 ± 14.0	0.16	0.13	22.3 ± 28.7	14.3 ± 12.8	0.01	0.46
Home SBP ARV (mmHg)	9.1 ± 4.3	8.5 ± 3.7	0.14	0.15	10.1 ± 5.0	8.4 ± 3.5	< 0.01	0.41	9.2 ± 4.4	8.7 ± 3.7	0.15	0.13	9.2 ± 4.6	8.9 ± 4.1	0.55	0.07
Home DBP ARV (mmHg)	6.4 ± 2.9	6.2 ± 2.6	0.40	0.09	6.7 ± 3.5	6.2 ± 2.4	0.06	0.20	6.5 ± 3.1	6.2 ± 2.2	0.20	0.11	6.3 ± 2.7	6.4 ± 2.8	0.83	−0.02
Office SBP ARV (mmHg)	14.3 ± 6.0	13.5 ± 5.2	0.24	0.13	15.2 ± 5.7	13.5 ± 5.8	< 0.01	0.30	14.8 ± 6.3	12.8 ± 4.7	< 0.01	0.35	15.3 ± 6.1	13.8 ± 5.7	0.04	0.25
Office DBP ARV (mmHg)	9.6 ± 4.1	9.7 ± 3.7	0.87	−0.02	9.8 ± 3.9	9.5 ± 4.0	0.49	0.07	9.9 ± 4.1	9.1 ± 3.7	0.05	0.19	10.6 ± 4.1	9.3 ± 3.9	0.01	0.34

*Note:* Values are presented as mean ± SD or *n/N* (%). SMDs quantify imbalance independently of sample size. An SMD < 0.1 indicates negligible imbalance. *p* values were derived using the two‐sample *t*‐test (continuous) and the χ^2^/Fisher’s exact test (categorical).

For each medication class and outcome, two weighted linear regression approaches were fitted: (1) an IPTW‐adjusted model including only the exposure variable and (2) a doubly robust model that additionally adjusted for the corresponding mean BP, number of measurements, and baseline covariates. Robust variance estimators were used to calculate standard errors and 95% confidence intervals (CIs). Additional analyses estimated the association between medication class and BP control status per ESH and ACC/AHA home and office BP targets, using IPTW‐weighted Poisson regression with robust standard errors to derive relative probability and 95% CIs.

To address potential residual confounding and combination‐therapy effects, three prespecified sensitivity analyses were performed. First, a true‐monotherapy subcohort analysis restricted to participants exposed to exactly one of the four focal classes and no non‐DHP CCB, using multivariable linear regression with HC3 robust standard errors. Second, class‐by‐class interaction tests for RAASi × DHP‐CCB, DHP‐CCB × diuretic, and RAASi × diuretic, fitted as IPTW‐weighted linear models with an interaction term and HC3 robust standard errors. Third, standardized effect sizes (Cohen’s d) for the primary doubly robust estimates against the pooled outcome standard deviation. Detailed specifications and results are reported in the Supporting Information.

All statistical analyses were conducted in R Version 4.3.1. A two‐sided *p* < 0.05 was considered statistically significant.

## 3. Results

Among the 495 participants, exposure frequencies were 77.6% for RAASi 384, 34.3% for beta‐blockers 170, 62.0% for DHP‐CCBs 307, and 21.0% for diuretics 104. Baseline characteristics showed clinical imbalances with treatment selection (Table 1). Patients treated with beta‐blocker were older (60.8 ± 13.3 vs 54.5 ± 13.0 years) and had a higher prevalence of heart failure (15.9% vs 4.0%) and chronic kidney disease (16.5% vs 3.4%). Patients treated with DHP‐CCB had a higher office SBP (138.6 ± 12.2 vs 133.7 ± 13.4 mmHg). Among the 104 diuretic users, loop or potassium‐sparing agents were the most commonly prescribed subtype (*n* = 66, 63.5%), followed by hydrochlorothiazide (*n* = 29, 27.9%) and chlorthalidone (*n* = 15, 14.4%); subtype categories were not mutually exclusive.

**FIGURE 1:**
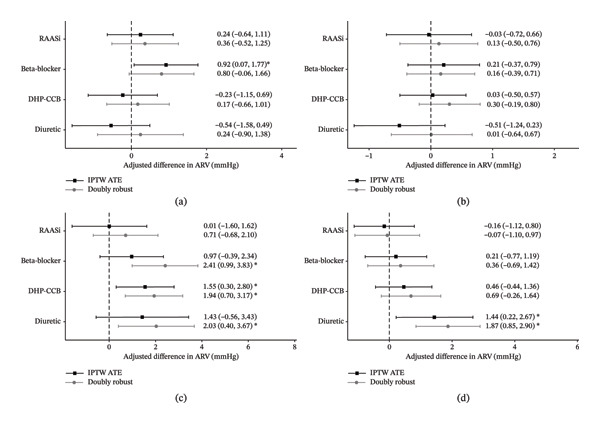
Class‐specific associations of antihypertensive medication with home and office BP variability. Forest plots illustrate adjusted mean differences in average real variability (ARV, mmHg) comparing exposure to each medication class versus nonexposure. Panels show (A) home SBP ARV, (B) home DBP ARV, (C) office SBP ARV, and (D) office DBP ARV. Gray circles and bars indicate inverse probability of treatment‐weighted (IPTW) average treatment effect estimates. Black squares and bars indicate doubly robust estimates (IPTW plus covariate adjustment). Horizontal bars represent 95% confidence intervals, and the vertical dashed line marks no difference (0 mmHg). Positive values indicate higher variability with exposure. Asterisks indicate confidence intervals that exclude zero (*p* value < 0.05). Numeric values adjacent to each estimate denote the point estimate with its 95% confidence interval. RAASi, renin–angiotensin system inhibitor; DHP‐CCB, dihydropyridine calcium channel blocker.

For systolic ARV, the doubly robust estimate for beta‐blockers suggested a modest increase (+0.92 mmHg; 95% CI, 0.07–1.77) (Table 2, Figure 1). RAASi, DHP‐CCBs, and diuretics showed estimates near zero. These findings indicate limited class differentiation in home BP variability, with only a small, directionally higher home SBP variability signal for beta‐blockers.

**TABLE 2:** Blood pressure variability by medication class for systolic and diastolic ARV.

Exposure	Outcome	Adjustment	Estimate (confidence interval)
*Home BP ARV*			
RAASi	SBP	IPTW ATE	0.36 (−0.52–1.25)
		Doubly robust	0.24 (−0.64–1.11)
	DBP	IPTW ATE	0.13 (−0.50–0.76)
		Doubly robust	−0.03 (−0.72–0.66)

Beta‐blocker	SBP	IPTW ATE	0.80 (−0.06–1.66)
		Doubly robust	0.92 (0.07–1.77)
	DBP	IPTW ATE	0.16 (−0.39–0.71)
		Doubly robust	0.21 (−0.37–0.79)

DHP‐CCB	SBP	IPTW ATE	0.17 (−0.66–1.01)
		Doubly robust	−0.23 (−1.15–0.69)
	DBP	IPTW ATE	0.30 (−0.19–0.80)
		Doubly robust	0.03 (−0.50–0.57)

Diuretic	SBP	IPTW ATE	0.24 (−0.90–1.38)
		Doubly robust	−0.54 (−1.58–0.49)
	DBP	IPTW ATE	0.01 (−0.64–0.67)
		Doubly robust	−0.51 (−1.24–0.23)

*Office BP ARV*			
RAASi	SBP	IPTW ATE	0.71 (−0.68–2.10)
		Doubly robust	0.01 (−1.60–1.62)
	DBP	IPTW ATE	−0.07 (−1.10–0.97)
		Doubly robust	−0.16 (−1.12–0.80)

Beta‐blocker	SBP	IPTW ATE	2.41 (0.99–3.83)
		Doubly robust	0.97 (−0.39–2.34)
	DBP	IPTW ATE	0.36 (−0.69–1.42)
		Doubly robust	0.21 (−0.77–1.19)

DHP‐CCB	SBP	IPTW ATE	1.94 (0.70–3.17)
		Doubly robust	1.55 (0.30–2.80)
	DBP	IPTW ATE	0.69 (−0.26–1.64)
		Doubly robust	0.46 (−0.44–1.36)

Diuretic	SBP	IPTW ATE	2.03 (0.40–3.67)
		Doubly robust	1.43 (−0.56–3.43)
	DBP	IPTW ATE	1.87 (0.85–2.90)
		Doubly robust	1.44 (0.22–2.67)

*Note:* Weighted linear models estimate the average treatment effect (ATE) of each medication class on ARV (mmHg). Doubly robust models further adjust for covariates, hospital fixed effects, the corresponding mean blood pressure, and the number of measurements.

In contrast, office ARV demonstrated clear class separation. For systolic ARV, IPTW ATE estimates were higher with beta‐blockers (+2.41 mmHg; 95% CI, 0.99–3.83), DHP‐CCBs (+1.94 mmHg; 95% CI, 0.70–3.17), and diuretics (+2.03 mmHg; 95% CI, 0.40–3.67). Doubly robust models attenuated the beta‐blocker effect (+0.97; 95% CI, −0.39–2.34), but DHP‐CCB remained elevated (+1.55 mmHg; 95% CI, 0.30–2.80). For diastolic ARV, diuretics were associated with higher office BP variability (IPTW, +1.87 mmHg; 95% CI, 0.85–2.90; doubly robust, +1.44 mmHg; 95% CI, 0.22–2.67). Drug class effects on office BP variability were more prominent than on home BP variability, suggesting an environment‐sensitive component (e.g., stress reactivity, clinic workflow, visiting spacing) that differs by class.

Next, BP control status by medication class was evaluated. Medication exposure was not associated with home BP control status according to either ESH (< 135/85 mmHg) or ACC/AHA (< 130/80 mmHg) guideline (Table 3). For office BP control status, DHP‐CCB exposure was associated with a lower probability of achieving the ACC/AHA target (< 130/80 mmHg; prevalence ratio (PR), 0.63; 95% CI, 0.41–0.96), with a similar trend for the ESH target (< 140/90 mmHg; PR, 0.82; 95% CI, 0.67–1.01). RAASi, beta‐blockers, and diuretic showed no significant difference. Thus, medication classes differ more in office‐setting target BP attainment than in home‐setting BP control, consistent with the ARV patterns.

**TABLE 3:** Blood pressure control status by medication status.

Exposure	BP control definition	Prevalence ratio (95% CI)
RAASi	Home BP < 135/85 mmHg (ESH criteria)	0.99 (0.87–1.14)
	Home BP < 130/80 mmHg (ACC/AHA criteria)	1.00 (0.77–1.29)
	Office BP < 140/90 mmHg (ESH criteria)	1.22 (0.91–1.65)
	Office BP < 130/80 mmHg (ACC/AHA criteria)	1.19 (0.69–2.05)

Beta‐blocker	Home BP < 135/85 mmHg (ESH criteria)	0.92 (0.79–1.07)
	Home BP < 130/80 mmHg (ACC/AHA criteria)	0.86 (0.66–1.13)
	Office BP < 140/90 mmHg (ESH criteria)	1.05 (0.83–1.33)
	Office BP < 130/80 mmHg (ACC/AHA criteria)	1.16 (0.74–1.83)

DHP‐CCB	Home BP < 135/85 mmHg (ESH criteria)	1.04 (0.91–1.19)
	Home BP < 130/80 mmHg (ACC/AHA criteria)	0.95 (0.75–1.21)
	Office BP < 140/90 mmHg (ESH criteria)	0.82 (0.67–1.01)
	Office BP < 130/80 mmHg (ACC/AHA criteria)	0.63 (0.41–0.96)

Diuretic	Home BP < 135/85 mmHg (ESH criteria)	0.93 (0.77–1.13)
	Home BP < 130/80 mmHg (ACC/AHA criteria)	0.93 (0.67–1.28)
	Office BP < 140/90 mmHg (ESH criteria)	1.02 (0.80–1.30)
	Office BP < 130/80 mmHg (ACC/AHA criteria)	1.37 (0.86–2.19)

*Note:* Values represent prevalence ratios with 95% confidence intervals (CI) for achieving blood pressure control, estimated using inverse probability of treatment weighting (IPTW) based on propensity scores. Blood pressure (BP) control was defined according to two international guideline thresholds: ESH criteria (home BP < 135/85 mmHg; office BP < 140/90 mmHg) and ACC/AHA criteria (home BP < 130/80 mmHg; office BP < 130/80 mmHg). RAASi, renin–angiotensin system inhibitor; DHP‐CCB, dihydropyridine calcium channel blocker.

Abbreviations: ACC/AHA, American College of Cardiology/American Heart Association; ESH, European Society of Hypertension.

To test whether white‐coat effect explains medication class differences, models included a continuous measure of white‐coat effect magnitude (centered difference between office and home mean BP) and a binary indicator of white‐coat effect based on European Society of Hypertension thresholds, in addition to prespecified baseline covariates and the number of BP measurements. Both white‐coat metrics were entered simultaneously in the same adjustment model; the continuous office‐minus‐home difference retains statistical power, while the guideline‐based binary indicator reflects the categorical white‐coat phenotype used in clinical practice. After the adjustment, home BP ARV became neutral across medication classes (Table 4). For office BP ARV, DHP‐CCB exposure still showed a higher systolic variability (+1.79 mmHg; 95% CI, 0.48–3.10), and diuretics showed a higher diastolic variability (+1.38 mmHg; 95% CI, 0.20–2.57).

**TABLE 4:** Association between antihypertensive medication class and BP variability after white‐coat effect adjustment.

Exposure	Outcome	Adjusted difference in ARV (mmHg) (95% CI)
RAASi	Home SBP ARV	−0.12 (−1.19–0.95)
	Home DBP ARV	−0.46 (−1.32–0.40)

Beta‐blocker	Home SBP ARV	0.67 (−0.15–1.50)
	Home DBP ARV	0.15 (−0.44–0.73)

DHP‐CCB	Home SBP ARV	−0.15 (−1.19–0.88)
	Home DBP ARV	0.01 (−0.56–0.59)

Diuretic	Home SBP ARV	−0.78 (−1.93–0.37)
	Home DBP ARV	−0.77 (−1.62–0.08)

RAASi	Office SBP ARV	0.01 (−1.63–1.66)
	Office DBP ARV	−0.26 (−1.18–0.67)

Beta‐blocker	Office SBP ARV	1.09 (−0.24–2.42)
	Office DBP ARV	0.33 (−0.65–1.32)

DHP‐CCB	Office SBP ARV	1.79 (0.48–3.10)
	Office DBP ARV	0.50 (−0.41–1.40)

Diuretic	Office SBP ARV	1.39 (−0.71–3.49)
	Office DBP ARV	1.38 (0.20–2.57)

*Note:* Values represent adjusted mean differences in average real variability (ARV, mmHg) with 95% confidence intervals (CIs), estimated using inverse probability of treatment weighting (IPTW) with covariate adjustment. Models included both a continuous measure of white‐coat effect magnitude (centered difference between office and home mean BP) and a binary indicator of white‐coat effect based on European Society of Hypertension thresholds, in addition to prespecified baseline covariates and the number of BP measurements.

Sensitivity analyses yielded findings largely consistent with the primary results, with one notable exception. In the true‐monotherapy subcohort, the class‐specific associations with home BP variability remained of similar direction and magnitude but with wider CIs, because monotherapy was sufficiently prevalent only for RAASi and DHP‐CCB to permit estimation (Supporting Table S2). Class‐by‐class interaction testing (Supporting Table S3) revealed a significant DHP‐CCB × diuretic interaction on home BP variability. The combination was associated with higher home systolic ARV (interaction term +3.43 mmHg, *p* = 0.001) and higher home diastolic ARV (+1.77 mmHg, *p* = 0.006) beyond the additive contribution of each class, whereas no significant interaction was observed on office BP variability, nor for RAASi × DHP‐CCB or RAASi × diuretic on any outcome. Standardized effect sizes (Cohen’s d) for the principal doubly robust estimates ranged from approximately 0.12 to 0.38, indicating small‐to‐modest magnitudes (Supporting Table S4).

## 4. Discussion

The present study offers three incremental contributions over the existing literature. First, by contrasting home and office BP variability within the same patients, it isolates a setting‐specific pattern that single‐setting studies cannot detect, namely, minimal class differentiation at home but pronounced differentiation in clinic. Second, by applying both continuous and guideline‐based white‐coat‐effect adjustments, it demonstrates that the clinic‐specific associations are not fully explained by white‐coat reactivity. Third, by focusing on short‐interval ARV rather than visit‐to‐visit SD, it highlights a variability phenotype that may be physiologically and mechanistically distinct from that emphasized in prior landmark trials.

Previously, the differential effects of antihypertensive drug classes on BP variability have been extensively studied in large‐scale trials, particularly focusing on visit‐to‐visit variability. The Anglo‐Scandinavian Cardiac Outcomes trial (ASCOT‐BPLA) demonstrated that amlodipine‐based regimens were associated with significantly lower clinic BP variability compared to atenolol‐based strategies, with this reduction partially explaining the stroke reduction observed in the CCB arm [15]. Similarly, the Antihypertensive and Lipid‐Lowering Treatment to Prevent Heart Attack trial (ALLHAT) showed that both chlorthalidone and amlodipine achieved lower visit‐to‐visit BP variability compared to Lisinopril [16]. Importantly, across multiple trials, the effects of treatment on both mean BP and BP variability accounted for treatment effects on stroke risk, with both parameters remaining independently significant in combined models.

A systematic review of several observational and small‐scale studies indicated that home BP variability was greater with beta‐blocker, that comparison between angiotensin receptor blockers (ARBs) and CCBs produced conflicting findings, and that the overall consistency of evidence was low [17]. Our finding of minimal drug class differences in home BP variability among patients receiving RAASi, beta‐blocker, DHP‐CCBs, or diuretics is consistent with results from the Hypertension Objective Treatment Based on Measurement by Electrical Devices of Blood Pressure (HOMED‐BP) trial [18]. In this randomized study of 2484 patients comparing CCBs, angiotensin‐converting enzyme (ACE) inhibitors, and ARBs, changes in home BP VIM and ARV did not differ significantly among drug classes after adjustment. These findings suggest that the controlled, stress‐free home environment may attenuate pharmacological class differences that are more apparent in clinic settings.

A novelty of the present study lies in the systemic comparison of drug class effects across measurement environments. While home BP variability remained largely class‐neutral, office BP variability demonstrated clear associations with specific drug classes—particularly higher ARV with DHP‐CCBs for SBP and diuretics for DBP. These office‐specific effects persisted after white‐coat effect adjustment, suggesting mechanisms beyond simple white‐coat reactivity. DHP‐CCB, as potent vasodilators, may trigger compensatory sympathetic activation that became more apparent under acute stress of clinic visits, manifesting as larger short‐interval oscillations despite effective mean BP reduction. Similarly, diuretics’ effects on volume status may contribute to greater BP fluctuations when office measurements are obtained at varying intervals relative to dosing or diuresis.

The apparent discrepancy between our office BP variability results and trial evidence suggesting CCB superiority requires careful interpretation, and a key consideration is the time scale of variability being captured. Landmark trials including ASCOT‐BPLA, ALLHAT, and the SPRINT substudy [19] examined visit‐to‐visit variability over months to years, whereas office ARV in the present study reflects reading‐to‐reading fluctuation within a clinic encounter, a much shorter time scale that is more sensitive to acute hemodynamic perturbations and less subject to long‐term vascular remodeling. Additional supporting evidence for short‐interval variability effects comes from Webb and colleagues, whose meta‐analysis demonstrated heterogeneous class effects on within‐visit variability [20], and from the ASCOT‐10 follow‐up by Gupta and colleagues, which reported that long‐term visit‐to‐visit BP variability remained an independent predictor of cardiovascular events even a decade after trial randomization [21]. Furthermore, there are substantial differences in BP measurement standardization between clinical trials and real‐world registries, with trials employing highly protocolized procedures while registry data inherit the variability of routine clinical practice. This dual effect would be consistent with our observation that home BP variability, measured in a more controlled and stress‐free environment, remained largely class‐neutral, while office measurements revealed context‐dependent physiological responses that are averaged out over longer observation periods in visit‐to‐visit analyses.

Our findings have several clinical implications. The class‐neutrality of home BP variability suggests that antihypertensive medication selection for home BP control should prioritize sustained BP lowering and adherence rather than specific class‐based BP variability. However, for patients with elevated office BP variability—particularly those on DHP‐CCBs or diuretics—targeted interventions may be considered. These could include previsit rest protocols, standardized measurement timing, checking home BP variability, or consideration of combination therapy with complementary classes. The persistence of class differences after white‐coat effect adjustment suggests that simple environmental modifications may be insufficient, requiring more comprehensive approaches. These recommendations align with the most recent hypertension guidelines, which reinforce out‐of‐office BP measurement and individualized BP targets, including the 2024 European Society of Cardiology guideline and the 2025 American Heart Association and American College of Cardiology guideline [22, 23].

An additional hypothesis‐generating observation emerged from exploratory class‐by‐class interaction tests. Concurrent use of DHP‐CCB and diuretic was associated with significantly greater home ARV than would be predicted by the sum of the two individual class effects (home SBP ARV interaction +3.43 mmHg, 95% CI 1.39 to 5.47, *p* = 0.001; home DBP ARV +1.77 mmHg, 95% CI 0.51 to 3.03, *p* = 0.006), whereas no such synergy was detected in the office setting. Although this interaction should be interpreted with caution because it derives from a subset of 55 concurrent users and was identified without formal multiplicity adjustment, the pattern is mechanistically plausible: DHP‐CCB‐induced vasodilation combined with diuretic‐induced volume fluctuation could theoretically amplify short‐term hemodynamic oscillations under stable home conditions. This preliminary signal warrants confirmation in larger datasets before any clinical inference is drawn.

Several limitations warrant consideration. First, the observational design precludes definitive causal inference despite propensity‐score weighting and doubly robust modeling. Because patients receiving beta‐blockers or diuretics were older and had a higher prevalence of heart failure, confounding by indication, through heart‐failure‐related sympathetic activation and age‐related arterial stiffness, may have contributed to the office BP variability associations; in sensitivity analyses excluding patients with heart failure or those aged 75 years or older, the office‐specific associations were preserved (Supporting Tables S5 and S6). Second, the study was conducted at a single tertiary cardiology center, which may limit external generalizability to primary‐care populations. Third, antihypertensive exposure was assessed cross‐sectionally rather than as a longitudinal trajectory, so dose titration, class switching, and adherence over time could not be captured. Fourth, the diuretic class comprised heterogeneous agents (loop, potassium‐sparing, thiazide‐type) with distinct hemodynamic and neurohormonal profiles; although a subtype breakdown is provided in Supporting Table, sample sizes precluded class‐by‐subtype stratified modeling. Because thiazide‐type agents constituted a minority of diuretic users (Supporting Table S1), the higher office diastolic variability associated with diuretics may partly reflect loop or potassium‐sparing rather than thiazide agents. Fifth, home BP measurement technique and adherence were patient‐reported and may have introduced measurement noise that is not equally distributed across drug classes. Sixth, ambulatory BP monitoring data were not available, limiting our ability to characterize nocturnal or 24‐h variability phenotypes. Seventh, the observed between‐class differences were modest in absolute magnitude (generally < 1 mmHg in home ARV and 1 to 2 mmHg in office ARV), and whether such differences translate to clinically meaningful cardiovascular outcome differences requires prospective confirmation.

In conclusion, our findings demonstrate context‐dependent associations between antihypertensive drug classes and BP variability. Home BP variability was largely class‐neutral, whereas office BP variability differed between classes, with DHP‐CCBs and diuretics showing higher office ARV. These class differences persisted after white‐coat effect adjustment, suggesting mechanisms beyond simple clinic‐induced reactivity. For clinical decision‐making, home BP control should remain the principal therapeutic target. Attention to office BP variability and to potential class interactions may help identify patient subgroups warranting further evaluation, with prospective confirmation required in randomized or longitudinal studies.

## Funding

This work was supported by the MSIT (Ministry of Science and ICT), Korea, under the ICAN (ICT Challenge and Advanced Network of HRD) program (IITP‐2026‐RS‐2022‐00156439) supervised by the IITP (Institute of Information & Communications Technology Planning & Evaluation) and the Korean Society of Hypertension (grant number KSH‐R‐2023).

## Disclosure

All authors had full access to the data in this study and played a role in writing and critical revision of the manuscript.

## Conflicts of Interest

The authors declare no conflicts of interest.

## Supporting Information

Additional supporting information can be found online in the Supporting Information section.

## Supporting information


**Supporting Information** Supporting Table S1. Diuretic subtype distribution among diuretic‐exposed participants. Supporting Table S2. Class‐by‐class interaction tests for home and office average real variability. Supporting Table S3. True‐monotherapy sensitivity analysis for RAASi and DHP‐CCB. Supporting Table S4. Standardized effect sizes (Cohen’s d) for principal doubly robust estimates. Supporting Table S5. Sensitivity analysis excluding patients with heart failure. Supporting Table S6. Sensitivity analysis excluding patients aged 75 years or older.

## Data Availability

The data that support the findings of this study are available upon request from the corresponding author. The data are not publicly available due to privacy or ethical restrictions.
