# Supplementary material for: Differential Effects of Antihypertensive Drug Classes on Home and Office Blood Pressure Variability
Source: Int J Hypertens. 2026 Aug 2;2026:2633062. doi: 10.1155/ijhy/2633062 (PMC13430049; doi:10.1155/ijhy/2633062)
Supplement: Supplementary file 1 — Supporting Information Supporting Table S1. Diuretic subtype distribution among diuretic‐exposed participants. Supporting Table S2. Class‐by‐class interaction tests for home and office average real variability. Supporting Table S3. True‐monotherapy sensitivity analysis for RAASi and DHP‐CCB. Supporting Table S4. Standardized effect sizes (Cohen’s d) for principal doubly robust estimates. Supporting Table S5. Sensitivity analysis excluding patients with heart failure. Supporting Table S6. Sensitivity analysis excluding patients aged 75 years or older. [file IJHY-2026-2633062-s001.docx]

**Supplementary Materials**

*Differential Effects of Antihypertensive Drug Classes on Home and Office Blood Pressure Variability*

**Supplementary Table S1. Diuretic subtype distribution among diuretic-exposed participants.**

| **Subtype** | **N (cohort users)** | **% of full cohort (N=495)** | **% among diuretic users (n=104)** |
| --- | --- | --- | --- |
| Hydrochlorothiazide (HCT) | 29 | 5.9% | 27.9% |
| Chlorthalidone | 15 | 3.0% | 14.4% |
| Loop or potassium-sparing | 66 | 13.3% | 63.5% |

*Subtype categories are not mutually exclusive because some participants were prescribed more than one diuretic. Percentages are computed with N=495 (full analytic cohort) and n=104 (diuretic-exposed subset) as denominators. HCT, hydrochlorothiazide.*

**Supplementary Table S2. Class-by-class interaction tests for home and office average real variability.**

| **Class pair** | **Outcome** | **N total** | **N both** | **Interaction ∆ (95% CI), mmHg** | **p (interaction)** |
| --- | --- | --- | --- | --- | --- |
| RAASi × DHP-CCB | Home SBP ARV | 343 | 180 | 1.96 (-0.08 to 4.00) | 0.060 |
| RAASi × DHP-CCB | Home DBP ARV | 343 | 180 | 0.98 (-0.47 to 2.44) | 0.184 |
| RAASi × DHP-CCB | Office SBP ARV | 333 | 179 | 1.68 (-1.63 to 4.99) | 0.320 |
| RAASi × DHP-CCB | Office DBP ARV | 333 | 179 | 0.82 (-1.47 to 3.12) | 0.482 |
| DHP-CCB × Diuretic | Home SBP ARV | 343 | 55 | 3.43 (1.39 to 5.47) | 0.001 |
| DHP-CCB × Diuretic | Home DBP ARV | 343 | 55 | 1.77 (0.51 to 3.03) | 0.006 |
| DHP-CCB × Diuretic | Office SBP ARV | 333 | 61 | 0.40 (-2.30 to 3.10) | 0.771 |
| DHP-CCB × Diuretic | Office DBP ARV | 333 | 61 | 0.09 (-2.13 to 2.31) | 0.938 |
| RAASi × Diuretic | Home SBP ARV | 343 | 64 | 1.83 (-0.69 to 4.35) | 0.155 |
| RAASi × Diuretic | Home DBP ARV | 343 | 64 | 1.76 (0.10 to 3.41) | 0.037 |
| RAASi × Diuretic | Office SBP ARV | 333 | 71 | -2.88 (-7.90 to 2.14) | 0.261 |
| RAASi × Diuretic | Office DBP ARV | 333 | 71 | 0.41 (-2.65 to 3.48) | 0.791 |

*Interaction estimates derive from weighted linear models that include both class indicators and their product term; models are adjusted for the covariate set of the doubly robust primary analysis. N both denotes the number of participants concurrently exposed to both classes of the pair. P-values are two-sided and are not adjusted for multiple comparisons. ARV, average real variability; DBP, diastolic blood pressure; DHP-CCB, dihydropyridine calcium channel blocker; RAASi, renin-angiotensin system inhibitor; SBP, systolic blood pressure.*

**Supplementary Table S3. True-monotherapy sensitivity analysis for RAASi and DHP-CCB.**

| **Exposure (class monotherapy)** | **Outcome** | **Adjusted estimate (95% CI), mmHg** | **N (exposed / unexposed)** |
| --- | --- | --- | --- |
| RAASi | Home SBP ARV | 0.71 (-0.65 to 2.08) | 36 / 41 |
| RAASi | Home DBP ARV | 0.16 (-0.88 to 1.21) | 36 / 41 |
| RAASi | Office SBP ARV | -0.43 (-3.67 to 2.82) | 35 / 31 |
| RAASi | Office DBP ARV | 0.01 (-2.28 to 2.29) | 35 / 31 |
| DHP-CCB | Home SBP ARV | -0.73 (-2.13 to 0.67) | 28 / 49 |
| DHP-CCB | Home DBP ARV | -0.18 (-1.25 to 0.89) | 28 / 49 |
| DHP-CCB | Office SBP ARV | 0.85 (-2.65 to 4.34) | 20 / 46 |
| DHP-CCB | Office DBP ARV | 0.64 (-2.24 to 3.53) | 20 / 46 |

*The sensitivity cohort restricts exposure to participants on a single antihypertensive class (true monotherapy); unexposed participants are on other-class monotherapy. Adjusted estimates are obtained from multivariable linear models with heteroskedasticity-consistent (HC3) robust standard errors. Cell counts differ across outcomes because office BP and home BP were not available in every participant. Beta-blocker and diuretic monotherapy subgroups were too small (n<20) to support reliable estimation and are therefore not reported. ARV, average real variability; CI, confidence interval; DHP-CCB, dihydropyridine calcium channel blocker; HC3, heteroskedasticity-consistent type-3; RAASi, renin-angiotensin system inhibitor.*

**Supplementary Table S4. Standardized effect sizes (Cohen’s d) for principal doubly robust estimates.**

| **Exposure** | **Outcome** | **DR estimate (95% CI), mmHg** | **Pooled SD, mmHg** | **Cohen’s d** | **N** |
| --- | --- | --- | --- | --- | --- |
| Beta-blocker | Home SBP ARV | 0.92 (0.07 to 1.77) | 3.39 | 0.27 | 343 |
| Beta-blocker | Office SBP ARV | 0.97 (-0.39 to 2.34) | 5.59 | 0.17 | 333 |
| DHP-CCB | Office SBP ARV | 1.55 (0.30 to 2.80) | 5.59 | 0.28 | 333 |
| DHP-CCB | Office DBP ARV | 0.46 (-0.44 to 1.36) | 3.80 | 0.12 | 333 |
| Diuretic | Office DBP ARV | 1.44 (0.22 to 2.67) | 3.80 | 0.38 | 333 |

*Only class-outcome pairs with statistically significant doubly robust estimates (p<0.05) are listed. Pooled SD is the square root of the pooled within-group variance for each outcome. Cohen’s d = DR estimate / Pooled SD. Values of 0.20, 0.50, and 0.80 correspond to small, medium, and large effect sizes, respectively. ARV, average real variability; CI, confidence interval; DHP-CCB, dihydropyridine calcium channel blocker; DR, doubly robust; SD, standard deviation.*

**Supplementary Table S5. Sensitivity analysis excluding patients with heart failure.**

| **Exposure** | **Outcome** | **IPTW ATE (95% CI)** | **Doubly robust (95% CI)** |
| --- | --- | --- | --- |
| RAASi | Home SBP ARV | 0.28 (-0.62 – 1.19) | 0.17 (-0.72 – 1.07) |
| Beta-blocker | Home SBP ARV | 0.91 (-0.07 – 1.89) | 0.82 (-0.07 – 1.71) |
| DHP-CCB | Home SBP ARV | -0.10 (-0.99 – 0.80) | -0.61 (-1.59 – 0.37) |
| Diuretic | Home SBP ARV | 0.33 (-0.72 – 1.39) | -0.54 (-1.63 – 0.55) |
| RAASi | Home DBP ARV | 0.15 (-0.49 – 0.79) | -0.08 (-0.78 – 0.63) |
| Beta-blocker | Home DBP ARV | -0.04 (-0.69 – 0.61) | 0.22 (-0.36 – 0.81) |
| DHP-CCB | Home DBP ARV | 0.44 (-0.10 – 0.99) | -0.09 (-0.67 – 0.48) |
| Diuretic | Home DBP ARV | -0.15 (-0.85 – 0.55) | -0.51 (-1.25 – 0.23) |
| RAASi | Office SBP ARV | 0.58 (-0.92 – 2.07) | -0.17 (-1.79 – 1.45) |
| Beta-blocker | Office SBP ARV | 1.39 (-0.08 – 2.85) | 0.89 (-0.62 – 2.40) |
| DHP-CCB | Office SBP ARV | 1.72 (0.37 – 3.07) | 1.37 (0.00 – 2.74) |
| Diuretic | Office SBP ARV | 2.51 (0.30 – 4.72) | 1.43 (-0.95 – 3.80) |
| RAASi | Office DBP ARV | -0.07 (-1.15 – 1.02) | -0.18 (-1.14 – 0.78) |
| Beta-blocker | Office DBP ARV | -0.31 (-1.24 – 0.62) | 0.00 (-1.08 – 1.08) |
| DHP-CCB | Office DBP ARV | 0.78 (-0.20 – 1.76) | 0.54 (-0.42 – 1.51) |
| Diuretic | Office DBP ARV | 2.01 (0.72 – 3.30) | 1.11 (-0.28 – 2.50) |

Estimates are adjusted mean differences in average real variability (ARV, mmHg) with 95% confidence intervals, obtained after excluding participants with prior heart failure (analytic cohort n=395 for home and 351 for office BP). Models are identical to the primary analysis: inverse probability of treatment weighting (IPTW) for the average treatment effect, and a doubly robust specification additionally adjusting for the corresponding mean BP, the number of measurements, and baseline covariates, with robust standard errors. ARV, average real variability; CI, confidence interval; DBP, diastolic blood pressure; DHP-CCB, dihydropyridine calcium channel blocker; IPTW, inverse probability of treatment weighting; RAASi, renin-angiotensin system inhibitor; SBP, systolic blood pressure.

**Supplementary Table S6. Sensitivity analysis excluding patients aged 75 years or older.**

| **Exposure** | **Outcome** | **IPTW ATE (95% CI)** | **Doubly robust (95% CI)** |
| --- | --- | --- | --- |
| RAASi | Home SBP ARV | 0.05 (-0.80 – 0.89) | 0.10 (-0.76 – 0.96) |
| Beta-blocker | Home SBP ARV | 0.64 (-0.22 – 1.50) | 0.77 (-0.09 – 1.63) |
| DHP-CCB | Home SBP ARV | -0.15 (-1.00 – 0.71) | -0.59 (-1.55 – 0.37) |
| Diuretic | Home SBP ARV | 0.00 (-1.05 – 1.05) | -0.62 (-1.85 – 0.60) |
| RAASi | Home DBP ARV | 0.11 (-0.52 – 0.73) | -0.06 (-0.75 – 0.64) |
| Beta-blocker | Home DBP ARV | 0.12 (-0.43 – 0.68) | 0.22 (-0.41 – 0.85) |
| DHP-CCB | Home DBP ARV | 0.22 (-0.32 – 0.76) | -0.07 (-0.65 – 0.50) |
| Diuretic | Home DBP ARV | -0.11 (-0.76 – 0.55) | -0.56 (-1.44 – 0.31) |
| RAASi | Office SBP ARV | 0.49 (-1.00 – 1.97) | -0.06 (-1.74 – 1.62) |
| Beta-blocker | Office SBP ARV | 1.10 (-0.50 – 2.71) | 0.57 (-0.95 – 2.10) |
| DHP-CCB | Office SBP ARV | 1.67 (0.31 – 3.02) | 1.41 (-0.03 – 2.86) |
| Diuretic | Office SBP ARV | 2.53 (0.51 – 4.55) | 1.45 (-0.67 – 3.58) |
| RAASi | Office DBP ARV | -0.20 (-1.26 – 0.86) | -0.12 (-1.09 – 0.85) |
| Beta-blocker | Office DBP ARV | 0.16 (-0.88 – 1.21) | 0.03 (-1.01 – 1.07) |
| DHP-CCB | Office DBP ARV | 0.56 (-0.47 – 1.59) | 0.20 (-0.79 – 1.20) |
| Diuretic | Office DBP ARV | 2.22 (1.04 – 3.41) | 1.61 (0.28 – 2.94) |

Estimates are adjusted mean differences in average real variability (ARV, mmHg) with 95% confidence intervals, obtained after excluding participants aged 75 years or older (analytic cohort n=397 for home and 349 for office BP). Models are identical to the primary analysis as described for Supplementary Table S5. ARV, average real variability; CI, confidence interval; DBP, diastolic blood pressure; DHP-CCB, dihydropyridine calcium channel blocker; IPTW, inverse probability of treatment weighting; RAASi, renin-angiotensin system inhibitor; SBP, systolic blood pressure.
